# Supplementary material for: α-Tocotrienol and Redox-Silent Analogs of Vitamin E Enhances Bortezomib Sensitivity in Solid Cancer Cells through Modulation of NFE2L1
Source: Int J Mol Sci. 2023 May 27;24(11):9382. doi: 10.3390/ijms24119382 (PMC10253528; doi:10.3390/ijms24119382)
Supplement: Supplementary file 1 [file ijms-24-09382-s001.zip › ijms-2403923-supplementary.pdf]

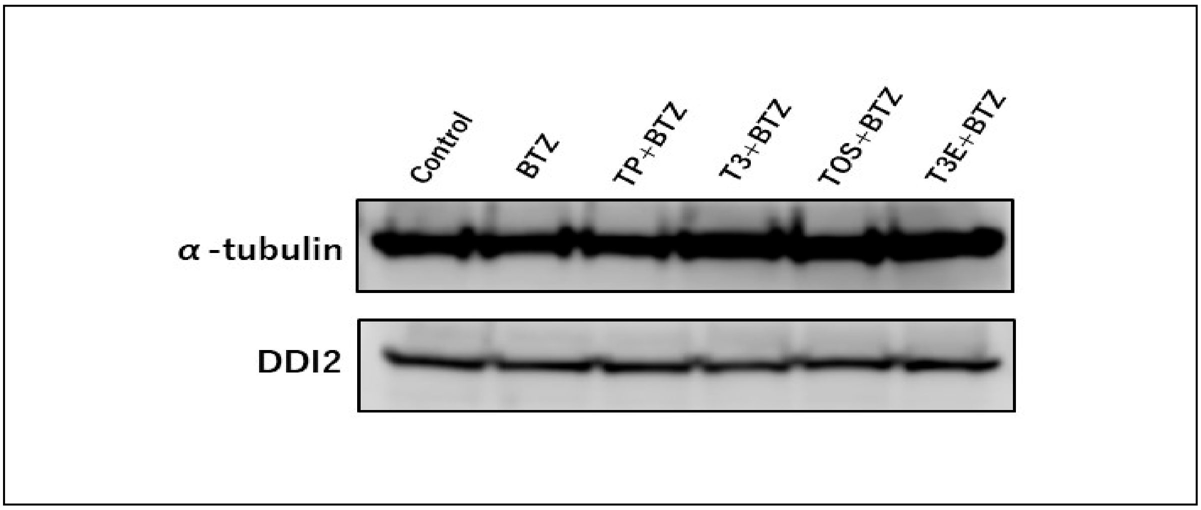

Figure S1. Effect of vitamin E alone on proteasome activity.
H2452 cells were treated with bortezomib 50 nM, TP 20 μM, T3 20 μM, TOS 20 μM, and, T3E 20 μM for 12 h. After the treatment, ubiquitinated protein levels in each sample were assessed by immunoblotting. α-Tubulin protein levels served as the loading control. Results are representative of three independent experiments. BTZ; Bortezomib, TP; α-tocopherol, T3; α-tocotrienol, TOS; α-tocopheryl succinate, T3E; 6-O-Carboxypropyl-alpha-tocotrienol.
